# 632 Ketamine as Prophylaxis for Depression and Post-Traumatic Stress Disorder Post-Burn Injury

**DOI:** 10.1093/jbcr/iraf019.261

**Published:** 2025-04-01

**Authors:** Davon Lee, Samrawit Zinabu, Alexis Edmonds, Emmanuel Ocampo, Tia Tyson, Sierra Lyles, Shaquan Taylor, Shivani Waghmare, Jerome Watts, Peyton Smith, Miriam Michael

**Affiliations:** Howard University College of Medicine and Burn Center at MedStar Washington Hospital Center; Howard University Hospital; Howard University College of Medicine; Howard University College of Medicine; Howard University College of Medicine; Howard University College of Medicine; Howard University College of Medicine; Howard University College of Medicine; Howard University College of Medicine; Howard University College of Medicine; Howard University College of Medicine

## Abstract

**Introduction:**

Ketamine is being used to treat patients for depression and has been studied in burn patients for that indication. Mental health issues are common after burns and evaluating ketamine use in prevention can provide valuable clinical insights. This study aims to explore ketamine’s efficacy in preventing depression and Post-Traumatic Stress Disorder (PTSD) in burn patients.

**Methods:**

This is a retrospective cohort study that used de-identified electronic medical records from a global research network. The study included burn patients 12-90 years of age, with third degree burns covering a minimum of 10% of total body surface area (TBSA). Patients were divided into two groups: those who received ketamine during their treatment and those who did not. ICD-10-CM codes were used to isolate patients with diagnosis of depression or PTSD spectrum disorders. Patients with those diagnosis prior to burn injuries were excluded. Characteristics of both cohorts were evened out with propensity score matching. Rates of depression and PTSD one year after the burn injury were then compared between the control and experimental groups.

**Results:**

There was a total of 361,639 patients. There were 344,290 patients in the control group, and 17,349 in the experimental cohort. The analysis showed that patients who received ketamine had a higher risk of developing depression and PTSD, compared to those who did not receive the drug. The risk ratio was 2.285(95% CI: 2.166, 2.409). Similarly, the odds ratio was 2.661 (95% CI: 2.502, 2.831). Kaplan-Meier survival analysis demonstrated lower survival probability (fewer patients remaining free of depression) in the ketamine group (71.74%) compared to the control group (85.94%). The log-rank test exhibited statistically significant differences between the control group and patients who received ketamine (χ2 = 924.760, p = 0.000), with a hazard ratio of 2.370 (95% CI: 2.238 to2.510, p = 0.000).

**Conclusions:**

The data suggests that patients who received ketamine had a significantly higher risk of depression and PTSD than those who did not. Contrary to the hypothesis that ketamine may protect against psychiatric disorders, our findings suggest the opposite. The results challenge the idea of ketamine’s protective role in mental health following burn injuries and highlight the need for further research into pharmacological prophylactic measures that decrease post-burn depression and PTSD.

**Applicability of Research to Practice:**

Pharmacological interventions may have implications in reducing the incidence of psychiatric disorders after burn injuries.

**Funding for the Study:**

N/A

